# Complete Genome Sequences of Influenza A/H1N1 Strains Isolated from Patients during the 2013-2014 Epidemic Season in Finland

**DOI:** 10.1128/genomeA.01523-14

**Published:** 2015-03-12

**Authors:** Petri Jalovaara, Polina Mishel, Hannimari Kallio-Kokko, Miia Valkonen, Anu Kantele, Niina Ikonen, Ilkka Julkunen, Laura Kakkola, Anna Kutsaya, Tytti Vuorinen, Pirkko Mattila, Henrikki Almusa, Denis Kainov

**Affiliations:** a The Institute for Molecular Medicine Finland (FIMM), University of Helsinki, Helsinki, Finland; b Helsinki University Hospital Laboratory (HUSLAB), Helsinki, Finland; c Helsinki University Central Hospital (HUCS), Helsinki, Finland; d The National Institute for Health and Welfare (THL), Helsinki, Finland; e Department of Virology, University of Turku, Turku, Finland

## Abstract

Here, we report 40 complete genome sequences of influenza A/H1N1 strains isolated from 33 nonhospitalized and 7 hospitalized patients during the 2013-2014 epidemic season in Finland. An analysis of the aligned sequences revealed no oseltamivir-resistant genotypes. As a whole, the recent viruses have drifted from the prototype A/California/7/2009 virus by ca. 1.3%.

## GENOME ANNOUNCEMENT

Influenza A viruses evolve rapidly and cause global pandemics and annual epidemics, which represent serious social and economic problems. The 2009 pandemic was caused by a novel influenza A virus of the H1N1 subtype. Since 2010, these viruses have contributed to seasonal epidemics. Approximately 20% of the general population becomes infected with these viruses, 17% of whom visit physicians. Of these patients, approximately 2% are hospitalized with more severe infections and resulting complications (1). Hospitalized patients often receive oseltamivir as an antiviral treatment.

Similarly to other influenza A viruses, the genomes of H1N1 viruses consist of 8 single-stranded RNA segments that encode 11 proteins: hemagglutinin (HA), neuraminidase (NA), nucleocapsid protein (NP), matrix (M) proteins (M1 and M2), nonstructural proteins (NS1), nuclear export protein (NEP), polymerase subunits (polymerase acidic [PA], polymerase basic 1 [PB1], and PB2), and auxiliary protein (PA-X). It was recently shown that a mutation in the NA gene of the 2009 pandemic virus (H275Y) is associated with resistance to oseltamivir (2).

Here, we collected nasopharyngeal aspirate samples from 7 hospitalized and 33 nonhospitalized patients diagnosed with influenza virus infections using reverse transcription-quantitative PCR (qRT-PCR) assays during the 2013-2014 epidemic season in Finland. The patients did not receive oseltamivir treatment before sampling. Due to a low concentration of viral RNA in some samples, we amplified the corresponding viruses in MDCK cell cultures. In total, 40 complete H1N1 genomes were sequenced using standard procedures (3). We translated the nucleotide sequences into amino acid sequences of 11 H1N1 proteins. An analysis of the aligned sequences revealed no H275Y mutation in NA associated with oseltamivir resistance. Thus, this and our previous efforts (3–5) revealed no oseltamivir-resistant genotypes in a total of 184 influenza H1N1 viruses isolated from patients from 2009 to 2014 in Finland.

### Nucleotide sequence accession numbers.

The full-genome sequences of influenza A viruses have been deposited in GenBank under the accession numbers KM366535 to KM366542, KM437714 to KM437721, KM437730 to KM437737, KM437738 to KM437745, KM437746 to KM437753, KM366583 to KM366590, KM366591 to KM366598, KM366599 to KM366606, KM437754 to KM437761, KM437762 to KM437769, KM366607 to KM366614, KM366615 to KM366622, KM366639 to KM366646, KM366623 to KM366630, KM366631 to KM366638, KM437802 to KM437809, KM437810 to KM437817, KM437834 to KM437841, KM366647 to KM366654, KM366655 to KM366662, KM437850 to KM437857, KM366663 to KM366670, KM366671 to KM366678, KM366679 to KM366686, KM366703 to KM366710, KM366711 to KM366718, KM366719 to KM366726, KM437634 to KM437641, KM366727 to KM366734, KM366735 to KM366742, KM366743 to KM366750, KM366751 to KM366758, KM366759 to KM366766, KM366767 to KM366774, KM366775 to KM366782, KM437642 to KM437649, KM437650 to KM437657, KM366375 to KM366382, KM366455 to KM366462, and KM366471 to KM366478.
